# Selections

**Published:** 1902-09

**Authors:** 


### SELECTIONS.

---

Alimentary Intoxication of the Pig. Professor Mathis believes that intoxication by the alimentary tract induced by changed foods is a common cause of death. By-products are largely employed in feeding pigs, such as refuse from the kitchen, potatoes not fit for human consumption, etc. Materials which cause no harm to ruminants, as mouldy corn, cotton cake, have a deleterious action upon the pig. It has been observed that soda present in slops used in washing out kitchen utensils and added to pig-wash has caused the death of pigs. The signs of intoxication are those of gastro-enteritis with nervous symptoms: vomition, tympany, diarrhœa, weakness of the hind quarters, spasm, convulsions. Prognosis is grave. Death may supervene in an hour, or after a few hours; those which live over the day generally recover, but are weak for some time. Treatment consists in giving large enemas repeatedly, and sulphate of soda in linseed tea. Milk and water alone should be allowed until the animals are well.—*Journ. de Med. Vet. et de Zoot. de Lyon; The Veterinarian*, July, 1902.

Goat's Milk. It would seem (*Therapeutic Gazette*) that goat's milk, which has for so long a time been rejected on account of its odor and composition, is about to be used much more extensively. Dr. Marfan has shown that in fresh milk there are certain zymoses which are destroyed by heat. The goat's milk does not contain any more casein than woman's milk, and according to Crepin's analysis the amount of casein and butter is about the same as in human milk. Dr. Boissard, obstetrician of the Paris hospitals, published last year a report on the results given by the use of goat's milk, and the latter were favorable. There is a special establishment in Paris where goats from the French and Swiss Alps are kept. The greatest cleanliness is observed, the jugs being washed in boiled water at milking time; the milkmen are obliged to wash their hands with soap; and the bottles and milk-cans are sterilized by being boiled in a solution of carbonate of sodium. It is a well-known fact that the goat does not readily contract tuberculosis, and this, of course, is a guarantee of some importance.—*The Medical Times*, May, 1902; *Modern Medicine*, July, 1902.

TRANSMISSION OF TUBERCULOSIS FROM MAN TO THE PIG. Tempel, the Director of the abattoir at Chemnitz, has established that tuberculosis of man may be transmitted to the pig by castration wounds, for he has seen several cases of primary tuberculosis of the scrotum and cord. He gives here four new cases, three in males, one in female. The cicatrices of castration contained caseous or calcareous tubercles. In one case the lesion formed a string along the cord up to the opening of the vas deferens into the urethra. The four animals had been operated upon by a castrator who for several years had scarcely been able to speak, and whose custom it was to place his bistoury in his mouth during his work. Similar cases have been recorded by John and Hohmuth.—*Zeitsch. f. Fleisch-Hygiene; the Veterinarian*, July, 1902.

VOMEERZIEKTE, OR VOMIT-SICKNESS OF SHEEP. This is a peculiar disease which affects sheep in certain localities of the districts of Hope Town, Carnarvon, Victoria West, Kenhardt, Prieska, Britstown, and portions of the adjoining districts. The general opinion entertained by the farmers respecting the originating cause of this disease, or functional derangement of the stomach, or of the nervous centre which regulates the spasmodic movements involved in the act of vomiting, is that it is due to a certain bush, known as the Vomeerboschje-geigeria passerinoides. This opinion, however, has not as yet been verified by experiment. In 1886 I fed some sheep with quantities of this plant obtained from a farm on which the disease was prevalent at that time, but I failed to produce any symptoms of the disease, and Veterinary Surgeon Dixon repeated this experiment in 1899 with the same negative result. It must be admitted, however, that this plant is generally found in considerable quantity on the veldt, where this disease is prevalent, and it may be that the poison is cumulative in character; hence stock may require to feed upon it for a considerable time before it develops its physiological effects. Another opinion is that this disease is due to the sheep eating a quantity of sand in their efforts to get at the young grass which springs up after the rain in the Karoo districts. This sand, they say, accumulates in the bowels, or rather at the pyloric or bowel end of the stomach, and blocks up the passage. I was, however, unable to confirm this opinion also by numerous post-mortem examinations. The immediate cause of death in the majority of the cases which I examined was bronchopneumonia, or inflammation of the lungs and bronchial tubes. This condition of the lungs is, however, a secondary effect of the primary irritation and the vomiting which supervenes. If the affected sheep are watched, more especially when following the flock, it will be observed that when they cough and vomit, they make an effort at the same time to swallow the food brought up, and a certain portion passes down the trachea into the lungs, where it causes acute inflammatory action. There is no doubt about this, as the portions of the vomited matter can easily be detected in the small bronchial tubes. I have little doubt, also, that this condition is greatly aggravated by driving the sick sheep with the flock, which increases the vomiting and distress very much.

*Post-mortem Appearances.* I made a post-mortem examination of several sheep in the early stages of the sickness, and found the lungs free from any appearance of irritation, nor was there any obstruction in the stomach or bowels, or accumulation of sand in any part of the intestinal tract.

*Symptoms.* The affected sheep walks behind the rest of the flock with a dull, tired, dejected appearance, and at short intervals it vomits. This spasmodic action is accompanied by a distressing sickly cough. There is not much food expelled from the mouth during the act of vomition, as the sheep makes an effort to retain it in the mouth and re-swallow it. When very sick the sheep does not attempt to feed, but will lie down almost constantly if left undisturbed.

*Cause.* There is very little doubt that this peculiar affection is due to some nervous irritant when the animal eats. Whether it is the vomeerboschje at present suspected, or some other plant, or some poisonous fungus that attacks certain plants at certain times of the year, which produces the physiological effects observed, I am unable to say; further experiments are necessary to determine these points.

*Treatment.* Many farmers successfully give lye, a solution made from the ash obtained from certain saline bushes, and used in the Colony for making soap. I have not experimented with this lye, but I have obtained very good results by giving bicarbonate of soda, a dessertspoonful; laudanum, one or two teaspoonsful; water, half a pint. Repeat this dose morning and evening. As soon as the vomiting has ceased give a dose of purgative medicine to clear out the offending food from the digestive organs, such as three to four ounces of Epsom salts in half a pint of warm water. The affected sheep should not be allowed to follow the flock, but should be kept in a quiet place, and disturbed as little as possible. They do not want to eat, and the stomach is much better to be kept undisturbed by any food until this irritation ceases.—D. HUTCHEON, Col. Veterinary Surgeon, *Agricultural Journal*, July, 1902.

THE PATHOLOGY OF CARCINOMA. H. Ribbert (*Deutsche med. Woch.*, Nov. 21, 1901) is of opinion that carcinomata are not due to a parasite, and gives his reasons for coming to this conclusion. He says that the production of carcinoma is supposed to depend on some stimulus which causes an overgrowth of cells of the epithelium.

This stimulus can only call forth changes of a character which are already present, and can never produce new qualities. There is no reason, however, to presuppose that this stimulus takes the form of a parasite. The parasitic theory claims that not only is the growth caused by the organism, but that its further development depends on the continued activity of the same. The process would correspond to a kind of symbiosis in which the living causal agent can only vegetate in epithelial cells. This would mean that the agent would damage the cells; but how a damaged cell would produce cell proliferation and not degenerative changes he cannot imagine. The term "carcinomatous degeneration" is clearly a misnomer. He pcints out that the parasite, once having attacked a cell, must produce its effect on that kind of cell only, and, in fact, be incapable of existing in any other cell. There would, therefore, have to be as many species of parasites as there are different kinds of cells. It is difficult to believe that this can in fact be so. Again, he points out a difficulty for the parasitic theory by saying that as soon as the original point of infection of the cancerous process is overstepped all further growth takes place solely from the cells already composing the cancer. According to the theory, other cells of the same histological character would have to be continually infected. He thinks that when the tumor grows in the immediate neighborhood of cells of the same character, if it depended on a parasitic invasion, these neighboring cells must of necessity be also invaded; but this, in fact, does not take place. He compares the pathological characters of a new-growth with those of an inflammatory growth, and claims from the comparison that the former begins as an inflammatory lesion of the connective tissue. Parasites could produce such a lesion, but they could not call forth the cell proliferation, which he explains mechanically. After following Czerny's arguments, and attempting to meet them in the light of his non-parasitic ideas, he concludes by disputing Behla's statements, that cancer is an infective process and that the increase of the disease can only be explained in this way, by stating that he doubts whether the increase actually is taking place; he considers that faulty diagnosis without the help of necropsies has much to answer for, and, even if there were a real increase, this might be explained by faulty hygienic conditions, etc., without the interpolation of parasites.—*British Medical Journal*, March 1, 1902; *Veterinary Journal*, May, 1902.

Bacterial Contamination of Milk. In order to determine the degree to which milk is necessarily contaminated during milking, and the extent of bacteriological growth which occurs during transit from the dairy to the city, Dr. W. H. Park, of New York (*Journal of Hygiene*, July, 1901, p. 391), undertook a series of investigations which yielded most striking and important results, which indicate that milk with far less bacterial contamination than is commonly the case can be supplied without appreciable increase of expense to the farmer and shipper. The milk of large cities comes necessarily from a wide extent of country, and part of it, at least, is, on arrival, already from twenty-four to forty-eight hours old, during which time the original bacterial content has enormously increased. One of the influences which determine the number of bacteria in the milk of the healthy cows, cleanliness in procuring, the degree to which it is cooled, and the length of time it is kept can be almost completely controlled. Reference is made to the inexcusable lack of cleanliness in the methods of procuring milk, and of care in cooling and keeping it during transportation, and to the unnecessary delay in shipping.

Milk obtained from cows where every reasonable means was taken to insure cleanliness, cooled within an hour to 45° F., and subsequently kept at that temperature, yielded after forty-eight hours on an average less than 11,000 bacteria per cubic centimetre. Taken during winter in well-ventilated, fairly clean, but dusty barns, and cooled within two hours to 45° F., the visible dirt having been cleaned off the hair of the udder; the milker's hands having been wiped off, but not washed; the milk pails and cans being clean, but the straining cloths dusty, it averaged, after forty-eight hours, 75,000 bacteria per cubic centimetre. Taken from more or less dirty cows stalled in ordinary barns, the teats having been cleaned slightly in the usual way by running the unwashed hands over them once before milking, it averaged, after forty-eight hours in winter, and in warm weather, respectively, 210,000 and 680,000 bacteria per cubic centimetre. Twenty samples of ordinary city milk taken immediately on arrival, many of them having been transported over 200 miles, yielded from 52,000 to 35,200,000 bacteria per cubic centimetre. Milk, as sold in the shops, yielded the following average figures: From the poorer tenement districts in midwinter, 1,977,692; in September, 15,163,600. From the more well-to-do districts in midwinter, 327,500; in September, 1,061,400. The influence of temperature was well shown by the results obtained on allowing portions of the same specimen to stand at different temperatures. Kept for twenty-four hours at temperatures below 50° F., no marked change occurred in the number of bacteria (3000) originally present per cubic centimetre; but at higher temperatures most astonishing growths occurred. Thus, at 60° F., 180,000; at 68° F., 450,000; at 86° F., 1,400,000,000, and at 94° F., no less than 25,000,000,000 per cubic centimetre. At the end of forty-eight hours the specimen kept at 60° F. had increased its bacterial content from 180,000 to 28,000,000, and that at 68° F. from 450,000 to 25,000,000,000. Those kept at the lower temperatures had not materially changed.

Park concludes that the most deleterious changes which occur in milk are not those due to ordinary adulteration, but are those produced by the multiplication of bacteria; and holds that it is the duty of health authorities to prevent the sale of milk rendered unfit for use through the bacteria and their products, and gradually to force the farmers and milkmen to use cleanliness, cold, and dispatch in the handling of their milk. As to standards, he believes in beginning with one of 500,000 for milk on entrance, and 1,000,000 on delivery. These figures are conceded to be ten times as high as milk should show, but by the enforcement of such a standard the farmer would receive a compulsory education in the necessity of preventing the presence and multiplication of bacteria.—*American Journal of the Medical Sciences*, May, 1902.

Influence of Alcohol upon the Evolution of Tuberculosis. Roos separated six guinea-pigs inoculated with tuberculosis into two groups. Three received the usual diet; the other three received, in addition, 25 c.c. of wine per kilo of weight daily. The average duration of the disease was ninety-four days for the former and ninety-five days for the latter.

Alcohol in the form of wine, even in large doses, did not seem to hasten the development of experimental tuberculosis in the guinea-pig.—*Rec. de Med. Vet.*

